# Optimal virtual monoenergetic image in “TwinBeam” dual‐energy CT for organs‐at‐risk delineation based on contrast‐noise‐ratio in head‐and‐neck radiotherapy

**DOI:** 10.1002/acm2.12539

**Published:** 2019-01-28

**Authors:** Tonghe Wang, Beth Bradshaw Ghavidel, Jonathan J. Beitler, Xiangyang Tang, Yang Lei, Walter J. Curran, Tian Liu, Xiaofeng Yang

**Affiliations:** ^1^ Department of Radiation Oncology and Winship Cancer Institute Emory University Atlanta GA 30322 USA; ^2^ Department of Radiology and Imaging Sciences and Winship Cancer Institute Emory University Atlanta GA 30322 USA

**Keywords:** contour delineation, dual energy CT, head‐and‐neck, radiation therapy, virtual monoenergetic image

## Abstract

**Purpose:**

Dual‐energy computed tomography (DECT) using TwinBeam CT (TBCT) is a new option for radiation oncology simulators. TBCT scanning provides virtual monoenergetic images which are attractive in treatment planning since lower energies offer better contrast for soft tissues, and higher energies reduce noise. A protocol is needed to achieve optimal performance of this feature. In this study, we investigated the TBCT scan schema with the head‐and‐neck radiotherapy workflow at our clinic and selected the optimal energy with best contrast‐noise‐ratio (CNR) in organs‐at‐risks (OARs) delineation for head‐and‐neck treatment planning.

**Methods and materials:**

We synthesized monochromatic images from 40 keV to 190 keV at 5 keV increments from data acquired by TBCT. We collected the Hounsfield unit (HU) numbers of OARs (brainstem, mandible, spinal cord, and parotid glands), the HU numbers of marginal regions outside OARs, and the noise levels for each monochromatic image. We then calculated the CNR for the different OARs at each energy level to generate a serial of spectral curves for each OAR. Based on these spectral curves of CNR, the mono‐energy corresponding to the max CNR was identified for each OAR of each patient.

**Results:**

Computed tomography scans of ten patients by TBCT were used to test the optimal monoenergetic image for the CNR of OAR. Based on the maximized CNR, the optimal energy values were 78.5 ± 5.3 keV for the brainstem, 78.0 ± 4.2 keV for the mandible, 78.5 ± 5.7 keV for the parotid glands, and 78.5 ± 5.3 keV for the spinal cord. Overall, the optimal energy for the maximum CNR of these OARs in head‐and‐neck cancer patients was 80 keV.

**Conclusion:**

We have proposed a clinically feasible protocol that selects the optimal energy level of the virtual monoenergetic image in TBCT for OAR delineation based on the CNR in head‐and‐neck OAR. This protocol can be applied in TBCT simulation.

## 1 INTRODUCTION

Dual‐energy CT (DECT) has been an important imaging modality with multiple clinical applications in radiology, including bone removal in angiography,^1^, ^2^, ^3^, ^4^ assessment of myocardial blood supply,^5^, ^6^, ^7^ renal calculi characterization,^8^, ^9^ and diagnosis of gout.^10^, ^11^ In these applications, DECT provides material specific information which is extracted by processing the projection datasets of two different energy spectra acquired by DECT. For example, bone and iodine contrast can be distinguished and mapped separately in DECT by utilizing their different energy dependences of attenuation, even if their Hounsfield Units (HUs) highly overlap each other.^12^, ^13^, ^14^

In addition to these material‐specific images, DECT is also able to synthesize virtual monoenergetic images.^15^ In conventional polychromatic CT images, beam‐hardening artifacts are commonly seen as cupping artifacts all over the images or bright/dark streaks between two high attenuation objects. These artifacts are caused by the polychromatic x rays which do not follow exponential attenuation as monochromatic x rays assumed in image reconstruction. Real monoenergetic images require scanning by monochromatic x‐ray sources usually generated by a large synchrotron, and is unavailable for clinical use. DECT has been proven in principle to be able to generate virtual monoenergetic images at a range of energies free of beam‐hardening artifacts since the DECT technique was first introduced.^16^ In addition, clinical studies have shown that monoenergetic images have comparable or even better noise level, contrast‐to‐noise ratio and CT number accuracy compared with conventional polychromatic images.^15^, ^17^, ^18^, ^19^

These material specific images and virtual monoenergetic images derived from DECT scans have already been utilized in routine diagnosis. It is now becoming attractive in radiation therapy (RT) with multiple potential applications proposed: including metal artifact reduction, normal tissue characterization, improved dose calculation, and functional imaging for target localization.^20^ However, such potential use of DECT in RT has been limited by two physical factors. The first factor is the concern of additional radiation dose of the DECT compared with conventional single‐energy CT. Additional dose was a concern when DECT was first simply implemented as two sequential single‐energy CT scans at two different x‐ray tube voltages without any optimization in scan protocol.^21^ With the introduction of novel implementations and dose reduction techniques, it has been reported that DECT can be routinely performed without additional dose or compromises in image quality.^22^, ^23^, ^24^ The second factor is the sub‐optimal implementations of DECT for RT. The two‐sequential‐scan scheme is greatly limited by its poor temporal coherence due to the time interval between two scans.^25^, ^26^ A more advanced and commonly used DECT implementation in diagnosis is dual‐source CT where two source‐detector pairs are mounted orthogonally to scan the same volume simultaneously with two different energy spectra.^27^, ^28^ However, the second detector is smaller in the limited space of CT gantry, resulting the field of view (FOV) usually limited to 35 cm.^29^

These drawbacks are overcome by “TwinBeam” DECT (TBCT) as the latest development in clinically available DECT systems.^30^ TBCT pre‐filters x rays from a single source with two different filters to split the x‐ray beam into two different energy spectra, as the corresponding halves of the detector capture them simultaneously.^31^, ^32^ The acquired low‐ and high‐energy dataset is then used for image reconstruction, material decomposition, and other DECT processing. Compared with other types of DECT, TBCT features for simultaneous DECT acquisition with a single set of x‐ray source and detector. TBCT has enabled routine use of DECT in radiation treatment planning since it has good temporal coherence, full FOV, and low hardware complexity and cost,^29^ although the energy separation of TBCT is inferior to other modalities mentioned above.^32^ To implement TBCT in RT workflow, it is essential to develop specific scan protocols to achieve optimal performance with relevant clinical studies.

TBCT, like other DECT systems, is able to generate virtual monoenergetic images between 40 and 190 keV. Such images with improved image quality are desirable to replace conventional single energy images in CT simulation. However, studies have shown that the image quality of the monoenergetic image depends on the selected energy; the noise level of the monoenergetic image has been shown to reach lowest value at certain energy depending on the object size, while image contrast keeps decreasing with energy increasing.^19^, ^33^, ^34^ Most of these studies are based on analytical derivation and phantom study focusing on diagnostic detectability, while such studies in RT are sparse. Image quality, such as contrast‐noise‐ratio (CNR), has been shown to be directly related to manual or automated tumor/organ‐at‐risk (OAR) delineation accuracy in CT simulation images,^35^, ^36^, ^37^, ^38^ thus the energy with optimal image quality for such clinical tasks in RT is worth investigation.

In this paper, we aim to investigate the optimal energy of virtual monoenergetic image in TBCT for OAR delineation of treatment planning based on CNR in head‐and‐neck (H&N) RT. We retrospectively investigated ten patients treated by H&N radiotherapy with DECT images acquired during simulation. We studied the image noise level and OAR contrast on monoenergetic images of different energies, and determined the energy at highest CNR value as optimal energy for each OAR.

## 2 MATERIALS AND METHODS

In this retrospective study, we analyzed the dataset of 10 patients with squamous cell carcinoma in H&N region. Patient selection standard is head and neck patients who were scanned in TBCT mode and had OARs delineated by physicians. The ten patients included six males and four females with ages ranging from 37 to 93. Their tumor sites vary from patient to patient including tonsil, buccal mucosa, tongue, and etc., and four patients underwent excisions. Each patient had CT simulation by TBCT in DECT mode with 110 s delay after 100 mL Omnipaque 300 iodine contrast injected at 2.5 mL/s, followed by treatment planning for RT. Institutional review board approval was obtained with no informed consent required for this HIPAA‐compliant retrospective analysis.

The DECT images were acquired using a Siemens SOMATOM Definition Edge TwinBeam CT scanner at 120 kVp with the patient in treatment position (pitch: 0.45, rotation time: 0.5 s, scan time: around 30 s, CTDIvol: around 20 mGy, reconstruction kernel: Q30f, tube current ranges from 500 to 650 mA, and metal artifact correction was in use). The 120 kVp x rays were split into high and low spectra by 0.05 mm tin and 0.6 mm gold filters, yielding high energy and low energy scans, respectively. These simultaneously acquired low‐ and high‐energy dataset were processed to synthesize monoenergetic images from 40 to 190 keV at 5 keV increments. Meanwhile, three other image sets were also derived from the same scan: composed polychromatic single energy CT images, mixed DECT images, and virtual non‐contrast (VNC) images. Composed images were reconstructed from raw projection dataset by disregarding spectral differences, that is, it is reconstructed as conventional single energy CT before any dual energy‐related process. Note that it is not a derivation from DECT. The composed images served as the treatment planning CT for tumor/OAR delineation and dose calculation. Mixed images were obtained as a linear combination of low‐ and high‐energy images with weightings recommended by vendor to simulate the standard 120 kVp images. VNC images were generated from low‐ and high‐energy dataset to removes iodine from contrast‐enhanced DECT images and reduce the need for an unenhanced CT scan.^39^ All of these three image sets were evaluated along with monoenergetic images in our study for comparison. The images were reconstructed by Siemens Syngo CT VA48A with spacing 1.27 × 1.27 × 1.5 mm^3^.

The virtual monoenergetic images were generated by monoenergetic plus algorithm (mono+) integrated in the Syngo software.^40^, ^41^ In mono+, the virtual monoenergetic image sets are generated using the high and low energy images from the dual‐energy exam through (a) basis material decomposition and (b) image synthesis using the determined mass density of the basis materials and monoenergetic linear attenuation coefficients. Compared with previously developed methods, mono+ uses a spatial frequency split approach to combine low frequency information from the low energy virtual monoenergetic image with the high frequency information from a high energy image. The resulting images have advantages of lower noise attributed from high energy images.

**Figure 1:**
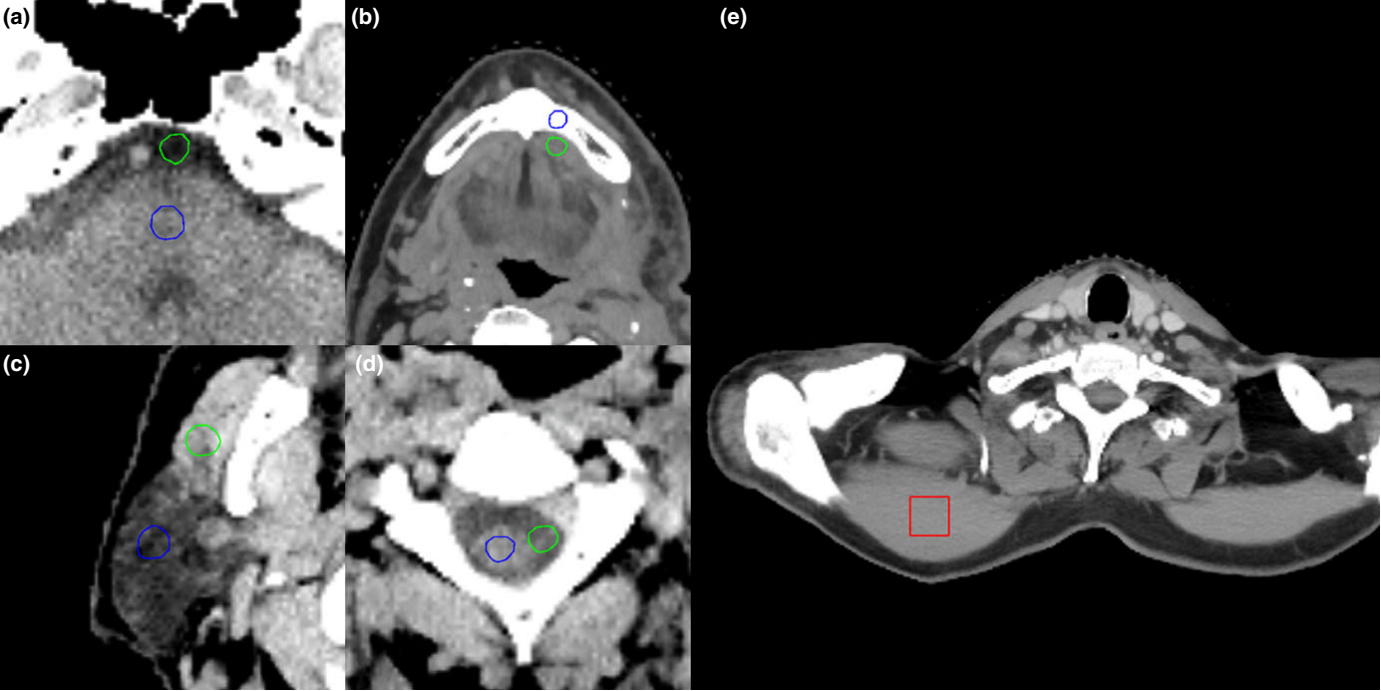
An example of contrast‐noise‐ratio calculation. Mean Hounsfield unit values are calculated within the ROIs of (a) brainstem (b) mandible, (c) parotid, and (d) spinal cord in blue circles and green circles. The red square in (e) is the area for noise standard deviation calculation in the uniform region in muscle.

OARs were defined by physicians on composed images, and were transferred to other image sets. Four commonly used OARs were selected as examples in this study: brainstem, mandible, parotid gland, and spinal cord. Mandible, which has high contrast on CT images, is included to investigate the CNR dependence on energy on high contrast material since other OARs have low contrast. For each image set, CNR values of these OARs were evaluated to quantify the contour detection. CNR describes the ability of the OAR to be detected against its background, which is defined as the ratio of contrast over noise, that is,

(1) $$CNR=\frac{HU_{\mathrm{OAR}}-HU_{OAR\_margin}}{\mathrm{Noise}},$$

where $HU_{\mathrm{OAR}}$ and $HU_{OAR\_margin}$ was the mean HU value within OAR contour and its surrounding margin with width around 2 mm, respectively. The subtraction between where $HU_{\mathrm{OAR}}$ and $HU_{OAR\_margin}$ quantifies the contrast of that OAR against its background. Note that the contour of each OAR is defined by physicians in clinic. The accuracy of the contours was not validated in this work. To minimize errors in CNR due to possible contouring inaccuracies, we set our OAR ROIs as 1‐cm diameter sphere region within the original OAR contour, and OAR background ROIs as 1‐cm diameter sphere region outside but close to original OAR contours. The noise level was calculated as the standard deviation of HU values in a uniform region (2.5 × 2.5 × 2.5 cm^3^) within muscle for each patient.^42^ As an example, Fig. 1 shows the ROI set for brainstem, mandible, parotid and spinal cord, and the uniform area in muscle for noise calculation on a patient.

The CNR of different OARs at each energy level was calculated and normalized by maximum CNR to generate a serial of spectral curves for each OAR. Based on these spectral curves of CNR, the mono‐energy corresponding to the curve peak (max CNR) for each OAR of was identified as the optimal mono‐energy setting for the corresponding OAR. One‐sided *t*‐tests were implemented between the CNR of the proposed optimal energy and any other energy levels among all patients for each OAR to evaluate statistical significance.

## 3 RESULTS

**Figure 2:**
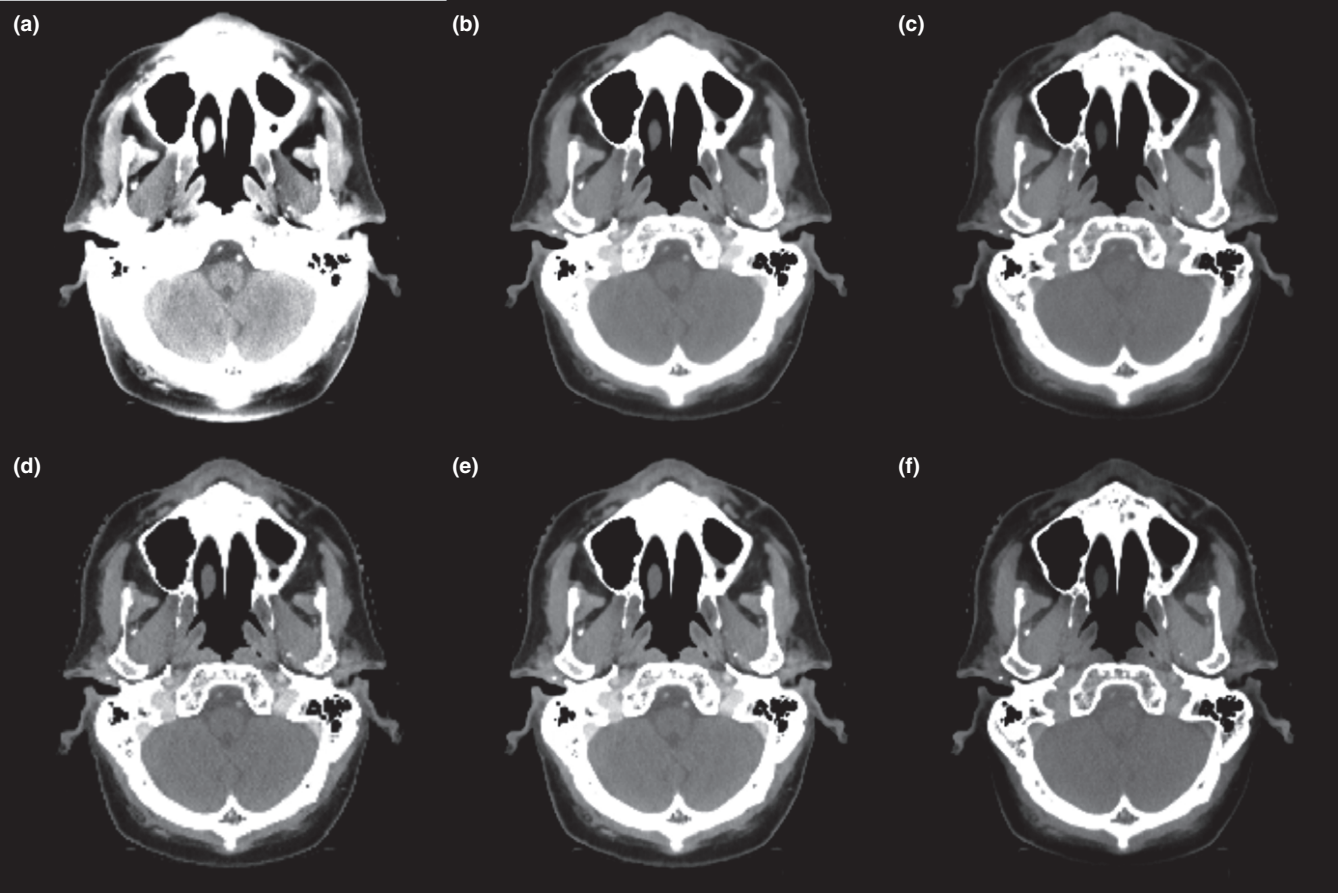
The axial view of monoenergetic images at (a) 40 keV, (b) 80 keV and (c) 190 keV as well as (d) composed, (e) mixed, and (f) virtual non‐contrast images from one patient as example. Display window: [−115, 180] HU.

Figure 2 showed a side‐by‐side comparison among the monoenergetic images at different energies as well as composed images, mixed, and VNC images from one patient as example. The image contrast increased with decreasing energy in monoenergetic images, as expected. The 40 keV monoenergetic image demonstrated the highest noise level and residual beam‐hardening artifacts around the skull. Composed and mixed images had similar contrast with slightly higher noise compared to the 80 keV monoenergetic image; VNC image had comparable contrast to the 190 keV monoenergetic image with higher noise.

**Figure 3:**
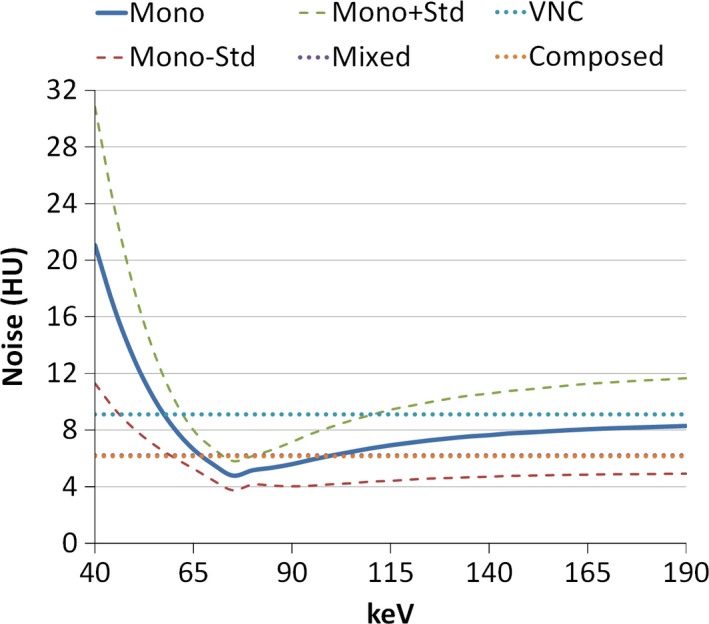
Mean noise level of monoenergetic images among ten patients changing with energy (solid line). The uncertainty is indicated by one standard deviation above and below mean value (dashed line). The noise level of composed, mixed, and virtual non‐contrast images are also shown in dotted lines.

Figure 3 demonstrated the noise level of monoenergetic images at different energies averaged among the ten patients. The noise level sharply decreased and then slowly increased to a stable value as energy increased, which is consistent with existing literatures.^15^, ^19^, ^43^, ^44^ The minimum noise was achieved around 80 keV. The noise levels of composed, mixed, and VNC images were all higher than the minimum noise of monoenergetic images (Fig. 3).

**Figure 4:**
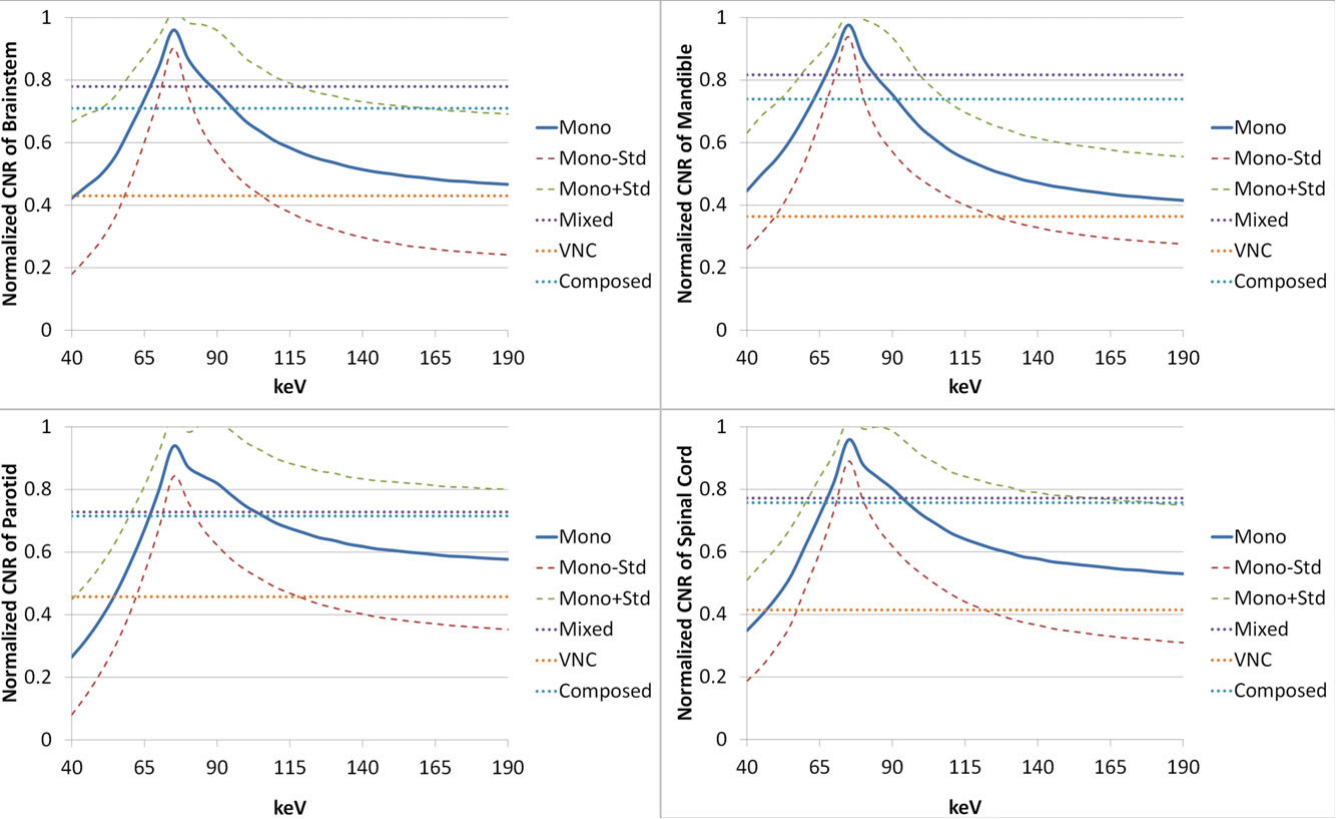
Mean contrast‐noise‐ratio (CNR) of monoenergetic images among ten patients changing with energy (solid line) in brainstem, mandible, parotid glands, and spinal cord. The uncertainty is indicated by one standard deviation above and below mean value (dashed line). The CNR of composed, mixed, and virtual non‐contrast images are also shown in dotted lines.

The mean CNR for each OAR among ten patients with monoenergetic images changing with energy were shown in Fig. 4. Peak CNR was approximately 80 keV for all OARs. Composed and mixed images exhibited approximately 70‐80% of the maximum CNR of monoenergetic images, while VNC images had only 40% of that. Quantitative results are summarized in Table 1. Overall, the optimal energy achieving maximum CNR for H&N cancer patients was 78.5 ± 5.0 keV, and this maximum CNR was higher than those on composed, mixed, and VNC images by 37%, 30% and 138%, respectively. One‐sided *t*‐tests were implemented between the CNR of the proposed 80 keV and any other energy levels among all patients for each OAR. The maximum *P*‐value is 0.037 < 0.05, which means that the CNR at the proposed 80 keV is higher than that at any other energy level with statistical significance.

**Table 1:** Optimal energy of maximum contrast‐noise‐ratio (CNR) in monoenergetic images. The *P*‐value indicates the maximum *P*‐value of CNR between the optimal energy and other energies. The maximum contrast and CNRs of monoenergetic images averaged among patients are shown in absolute value. The CNR of composed, mixed and VNC images are also listed as percentage of maximum CNR of monoenergetic images

OAR	Energy of maximum CNR (keV)	*P*‐value	Maximum contrast	Maximum CNR	% of max CNR in monoenergetic image		
	Mean ± SD		Mean ± SD	Mean ± SD	Composed	Mixed	VNC
Brain stem	78.5 ± 5.3	0.018	20.6 ± 12.5	2.5 ± 1.6	71.0%	78.0%	43.0%
Mandible	78.0 ± 4.2	0.001	1266.9 ± 336.3	156.1 ± 70.3	73.9%	81.7%	36.4%
Parotid	78.5 ± 5.7	0.023	27.8 ± 11.6	4.7 ± 1.7	71.5%	72.8%	45.7%
Spinal cord	78.5 ± 5.3	0.037	20.3 ± 10.5	2.9 ± 0.9	75.7%	77.2%	41.4%
Average	78.5 ± 5.0				73.0%	77.4%	41.6%

## 4 DISCUSSION

The introduction of TBCT enables DECT scans to be practical for routine use in RT. The benefits of TBCT when compared to prior generations of DECT include a 50‐cm field FOV and improved temporal coherence. Multiple potential applications of TBCT in RT have been proposed,^45^ which would assist in RT in terms of better simulation image quality and more accurate treatment plans. However, clinical validation and evaluation are required.

In this study, we have proposed an approach to select the optimal energy level of virtual monoenergetic images with maximum CNR for OAR delineation of H&N radiotherapy planning using TBCT. The average optimal energy was 78.5 ± 5.0 keV among the four OARs studied in this paper. These results suggested a TBCT simulation protocol for H&N cancer patients: patients are scanned by TBCT in DECT mode, and the acquired datasets are sent for reconstruction of both conventional composed images and 80 keV monoenergetic images. Contours of OARs are delineated on 80 keV images and propagated to composed images for treatment planning and dose calculation.

The 80‐keV protocol for H&N patients in this study may not be optimal for TBCT scans for other sites since it has been shown that the image quality of monoenergetic image depends on object size.^19^, ^33^, ^34^ More patients may be required for studies of other sites such as pelvis, due to the larger variation in object size among patient compared with H&N.

This study also provided valuable guidance in integrating TBCT fully into RT workflow. We demonstrated that the optimal virtual monoenergetic images by TBCT outperform conventional 120 kVp CT images in CNR of OARs by around 30%. To further exploit TBCT in RT, this study could be extended to other derived images for multiple clinical applications. For example, although the VNC image shows low CNR in this study, its removal of iodine may increase the dose calculation accuracy on contrast enhanced structures during treatment planning.^20^ A comprehensive study is needed to evaluate its dose calculation improvement compared with single energy CT.

We investigated the optimal energy of virtual monoenergetic image with the maximum CNR of OARs in TBCT in this study. Similar studies have been done on other DECT modalities. Pomerantz et al.^46^ reported 65–75 keV maximizing brain parenchymal image quality in GE Discovery CT750HD DECT scanner with fast‐kVp‐switching mode. For this GE scanner, Lam et al.^47^ demonstrated that 65 keV is optimal to achieve best signal‐to‐noise ratio for tissues in head and neck region. In head and neck region on dual‐source CT, Wichmann proposed 60 keV to improve lesion enhancement.^48^ Frellesen et al. recommended 40 keV to achieve best carcinoma‐to‐pancreas contrast on dual‐source CT, and Di Maso et al. found 40 keV to achieve both best carcinoma‐to‐pancreas contrast and CNR on TBCT.^45^, ^49^ Differences have been found among these studies due to different sites, imaging modalities and reconstruction method, and the metrics that each study optimized for are diverse, including signal‐to‐noise ratio, contrast, CNR, artifact index, and subjective rating. In this study, the proposed 80 keV is around where noise level is lowest (see Fig. 3), which means that noise plays a decisive role for maximizing the CNR in OAR at head and neck region. Similarly, in a series of studies by Leng et al. and Yu et al., a same trend of noise dependence on virtual energy is found.^15^, ^19^, ^43^ In these studies, the energies for lowest noise and for maximum CNR on iodine are both around 70 keV. These results indicate that the energy of lowest noise may determine the energy for maximum CNR in virtual monoenergetic images, and such energy may change with noise characteristics which highly depend on scan modality and reconstruction parameters.

In this paper, we did not investigate target CNR using TBCT. The reason is that among the patients studied in this paper, their treatment volumes were mainly defined by another imaging modality such as PET imaging, and they have very low contrast observed on CT images. Moreover, margins, nodes, and other surrounding tissues are usually included into treatment volume which contains multiple different materials.

In addition to CNR, other aspects of image quality determined by scan and reconstruction parameters may affect OAR delineation accuracy. Future studies could investigate the role of spatial resolution by different reconstruction kernel, pitch, and rotation time during scan in OAR delineation accuracy. Moreover, inter‐observer study could be included to determine images of optimal energy by quantifying contouring uncertainty among different physicians on same images, including overlapping percentage, volume changes and centroid differences.^50^ On the other hand, iodine contrast map derived from DECT scan has been shown to be useful for characterization of lesions in diagnosis,^51^ thus future work can be focused on its combination with CT and PET images for potential improvement in target delineation.

## 5 CONCLUSION

We have proposed a clinically feasible protocol that selects the optimal energy level of the virtual monoenergetic image in TBCT for head‐and‐neck to maximize CNR of OARs. The optimal energy was 80 keV in our clinic. This protocol can be applied in TBCT simulation.

## CONFLICT OF INTEREST

The authors declare no conflict of interest.
